# Mechanics of gelatin-based hydrogels during finite strain tension, compression and shear

**DOI:** 10.3389/fbioe.2022.1094197

**Published:** 2023-01-12

**Authors:** Hamid Yousefi-Mashouf, Lucie Bailly, Laurent Orgéas, Nathalie Henrich Bernardoni

**Affiliations:** ^1^ University Grenoble Alpes, Centre National de la Recherche Scientifique (CNRS), Grenoble INP, 3SR, Grenoble, France; ^2^ University Grenoble Alpes, Centre National de la Recherche Scientifique (CNRS), Grenoble INP, GIPSA-lab, Grenoble, France

**Keywords:** covalently cross-linked hydrogel, gelatin, glutaraldehyde, tunable mechanical properties, finite strains, vocal folds

## Abstract

**Introduction:** Among the biopolymers used to make hydrogels, gelatin is very attractive due to its biocompatibility, biodegradability and versatile physico-chemical properties. A proper and complete characterization of the mechanical behavior of these hydrogels is critical to evaluate the relevance of one formulation over another for a targeted application, and to optimise their processing route accordingly.

**Methods:** In this work, we manufactured neat gelatin and gelatin covalently cross-linked with glutaraldehyde at various concentrations, yielding to hydrogels with tunable mechanical properties that we characterized under finite strain, cyclic tension, compression and shear loadings.

**Results and Discussion:** The role of both the chemical formulation and the kinematical path on the mechanical performances of the gels is highlighted. As an opening towards biomedical applications, the properties of the gels are confronted to those of native soft tissues particularly complicated to restore, the human vocal folds. A specific cross-linked hydrogel is selected to mimic vocal-fold fibrous tissues.

## Introduction

Hydrogels are 3D networks of hydrophilic polymers able to absorb and hold a large amount of water without dissolving (*e.g.,* up to several hundred times their dry weight) (Mudiyanselage and Neckers, 2008; Burdick and Murphy, 2012; Zhang and Khademhosseini, 2017). Their softness and structural similarities with the extra-cellular matrix of human soft tissues make them materials of choice for biomedical applications (Lee and Mooney, 2001; Seliktar, 2012; Afewerki et al., 2019). Among the polymers used to form hydrogels, gelatin is very attractive due to its *in vivo* biocompatibility, biodegradability, versatile physico-chemical properties, and its abundance in renewable natural resources which allows for low-cost and eco-friendly implementations (Nur Hanani et al., 2014; Afewerki et al., 2019). Therefore, during the last decade, gelatin-based formulations have been proposed for electrospun fibres (Ratanavaraporn et al., 2010; Panzavolta et al., 2011; Kishan et al., 2015) and 3D scaffolds for tissue regeneration (Gomes et al., 2015; Hiwatashi et al., 2015; Poursamar et al., 2015; Kazemirad et al., 2016), microcarriers in drug delivery (Subramanian and Vijayakumar, 2013; Duconseille et al., 2015; Feyen et al., 2016; Heris et al., 2016; Imaizumi et al., 2021) and foams for wound dressing (Imani et al., 2013; Poursamar et al., 2016). More particularly, active research is underway to develop gelatin-based hydrogels to be injected into the vocal folds for surgical voice restoration (Heris et al., 2012; 2016; Kazemirad et al., 2016; Hiwatashi et al., 2017; Latifi et al., 2018; Ravanbakhsh et al., 2019; Imaizumi et al., 2021).

Gelatin is a hydrophilic protein fragment derived from collagen (Type I), that is the major fibrous structural protein in skin, bone and connective tissues of animals. Gelatin comes from the hydrolysis of the triple-helix structure of collagen, yielding to a randomly coiled structure. When cooling an aqueous solution of gelatin below ≈30–35°C, provided that the concentration is high enough (above ≈2% w/v), a thermo-reversible gel is formed by physical cross-linking, in particular due to partial recovery of the collagen helical structure (Bode et al., 2011; Gorgieva and Kokol, 2011; Dash et al., 2013; Xing et al., 2014; Campiglio et al., 2019). Gelation features of gelatin (*e.g.,* molecular weight, gel-forming temperature, chemical composition) depend on the collagen animal source (Michelini et al., 2020) or their processing route (Gorgieva and Kokol, 2011). Despite excellent physical and biochemical compatibilities, standard hydrogels based on neat gelatin present three main barriers to their potential applications: poor mechanical performances (*e.g.,* low elastic modulus, brittle failure), poor thermal stability in temperatures close to human body (*e.g.,* dissolution of the gel around 40°C), undesirable swelling under excessive hydratability, up to full disintegration into the solvent (Bigi et al., 2001; Hoffman, 2002; Farris et al., 2010; Dash et al., 2013). Such limitations can be overcome by promoting intermolecular associations along the gelatin amino acid sequences, and bonding gelatin polymer chains by covalent bonds. Among the possible candidates, glutaraldehyde (GA) allows to link together proteins *via* a high chemical reactivity towards NH_2_ groups, forming stable covalent bonds. GA is by far the most frequently used due to its low cost and efficiency in increasing the gel tensile strength, ductility as well as its denaturation temperature by a shift of ≈30°C (Bigi et al., 2001; Catalina et al., 2011; Poursamar et al., 2016). Although GA treatment is also known to leave cytotoxic residues, adverse effects can be minimized by using it in low concentrations: 0.05% v/v is reportedly enough to cross-link about 60% of gelatin amino groups (Bigi et al., 2001).

Faced with the growing need for such gelatin-based hydrogels and the proliferation of proposed formulations, characterization of their mechanical behavior becomes essential to understand the process/function relationships, to classify the added value of one formulation over another, and to evaluate its relevance for a targeted biomechanical application. Therefore, during the last decade, a few studies have investigated the mechanics of gelatin gels, (non)cross-linked with various reagents and shaped into various structures (films, foams or filled volumes):

• Some of these works have focused on single (shear or tensile) response of the gels using standard Dynamic Mechanical Analysis (DMA), *i.e.,* within the linear regime (Farris et al., 2011; Dash et al., 2013; Xing et al., 2014). These works allowed to quantify the shear (or tensile) dynamic moduli of the various formulations subjected to a frequency/temperature sweep. Typically, for neat gels, shear storage modulus (range of values 9–13 kPa) was reported one order of magnitude higher than the loss modulus, highlighting a predominant elastic response (Dash et al., 2013). Whatever the considered chemical cross-linkers (*e.g.,* functionalized cellulose nanowhiskers, 1-ethyl-3-(3-dimethylaminopropyl)-carbodiimide or GA-glycerol), their reaction induced an increase of the dynamic moduli by a ratio of 1 up to 100, depending on the degree of cross-linking.
• Other pioneer works have extended the field of study to large deformations in tension (Bigi et al., 2001; Farris et al., 2011; Poursamar et al., 2016) or in compression (Kwon and Subhash, 2010; Poursamar et al., 2016). These first results are sensitive to the gel processing route, yielding to reversed trends in some cases: considering air-dried films cross-linked with GA at several concentrations and immersed in a mixture of water and ethanol (Bigi et al., 2001), a significant stiffening was obtained even at low GA concentrations. However, the extensibility was here found to decrease while increasing GA concentration, and to reduce by about one order of magnitude with respect to that measured for uncross-linked films. Conversely, addition of GA in gelatin-pectin-glycerol films allowed to increase the tensile strength but also the elongation at break (by about 40%) (Farris et al., 2011). Finally, to our knowledge, a single study has characterized the mechanics of gelatin-based hydrogels in tension and compression so far, in the case of very specific porous scaffolds shaped by gas foaming (Poursamar et al., 2015).

In the end, the current experimental study of gelatin-based hydrogels is often limited to either a specific loading mode, with a single monotonic path to failure, or to standard infinitesimal strain analyses. These configurations are still far from those endured *in vivo* by living tissues, which are often subjected to many complex and coupled mechanical loadings upon finite strains and various strain rates. Therefore, this work aims to further investigate the mechanics of gelatin hydrogels under different loading modes (tension, compression, shear) and kinematics (finite strains, cyclic paths and various strain rates). Neat gelatin and gelatin cross-linked with GA of various concentrations were manufactured and characterized purposely. As an illustration and opening towards a current biomimetic challenge, we also confronted the mechanical performances of the gels to those of a native soft tissue particularly complicated to restore, namely the human vocal folds.

## Materials and methods

### Sample preparation

Pigskin gelatin powder (Type A, gel strength ≈300 g Bloom, Sigma-Aldrich®) and a glutaraldehyde mother solution (Grade I, 50% w/w in water, Sigma-Aldrich®) were used to produce the hydrogels. Two different processing routes were employed to elaborate samples made of neat gelatin (Ge) or gelatin cross-linked with glutaraldehyde (Ge-GA).

**Neat Ge hydrogels –** 30 mL of a gelatin aqueous solution (10% w/v) was obtained by dissolving 3 g of Ge powder in water for 30 min at 45°C (Portier et al., 2017). This concentration was chosen as an intermediate value based on previous studies dealing with gelatin-based hydrogels, reporting Ge concentrations within the range 2% w/v up to 20% w/v (Nichol et al., 2010; Bode et al., 2011; Farris et al., 2011; Pan et al., 2014; Poursamar et al., 2016; Goodarzi et al., 2019; Hipwood et al., 2022). Ultrapure water (18.2 MΩ) was used to minimize the non-uniform physical bonding network caused by unbalanced ionic charge distribution (Xing et al., 2014). The prepared solution was firstly homogenized using magnetic stirring (350 rpm). Then, it was poured into a customized Teflon® mold at room temperature (T ≈ 21°C) and relative humidity (RH ≈ 45%) for 1 h, and kept at 3°C for 24 h to form a rectangular gel plate (100 × 100 × 2 mm^3^). Finally, rectangular samples were cut from the plate at desired dimensions using two parallel razor blades, and marked with a random pattern made of small speckles for optical tracking during the mechanical tests.

**FIGURE 1:**
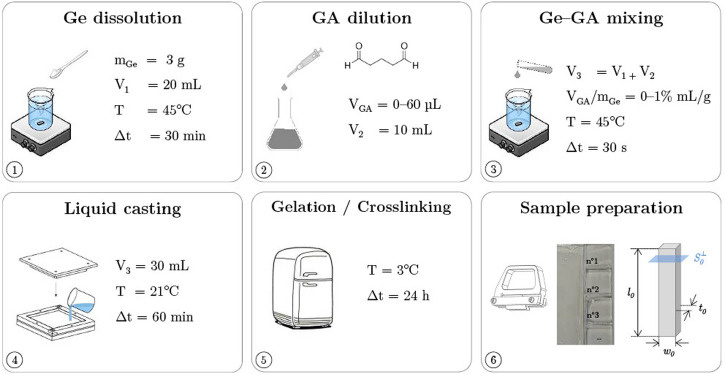
Processing route of gelatin hydrogels cross-linked with glutaraldehyde (Ge-GA samples).

**Ge hydrogels cross-linked with GA–**The preparation of cross-linked hydrogels comprised several steps (Figure 1). A gelatin aqueous solution was first prepared as described above, albeit for a smaller final volume (20 mL) and a higher gelatin concentration (15% w/v). In parallel, a given micro-volume V_*GA*_ of the GA mother solution was collected, and diluted in ultrapure water to prepare 10 mL of daughter solution. *V_GA_* was parametrically varied (15; 30; 45; 60 *μ*L) in order to manufacture samples with various degree of cross-linking. Ge and GA solutions were mixed together during 30 s at 45°C. The Ge-GA mixture (30 mL) was then casted into a rectangular mold to form a gel with a fixed concentration in gelatin (10% w/v), and a parametrical concentration of cross-linker so that *V_*GA*_/m_*Ge*_* ∈ [0.25%; 0.5%; 0.75%; 1%] mL/g. The steps of gelation in a cool atmosphere and shaping of samples were similar as for the neat Ge hydrogels. Note that for *V_*GA*_/m_*Ge*_* > 1% mL/g, the cross-linking kinetics was so fast that it prevented the castability of the Ge-GA mixture (see Supplementary Figure S1).

### Mechanical characterization

An experimental protocol was designed to characterise the finite-strain mechanics of Ge and Ge-GA samples under tension, compression and shear, as previously done on vocal-fold tissues (Cochereau et al., 2020).

**FIGURE 2:**
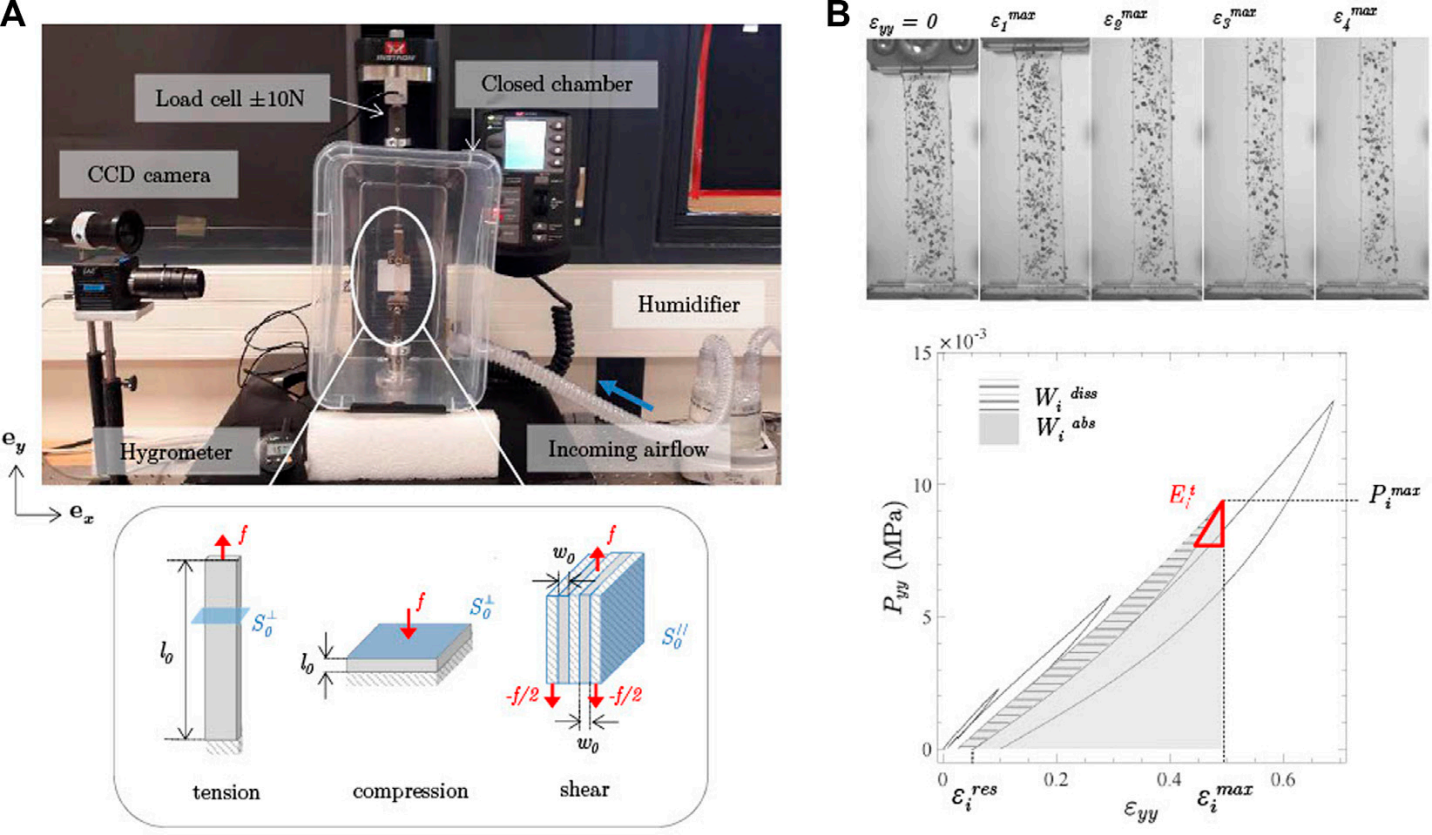
**(A)** Overview of the experimental set-up *(top)*, and schemes illustrating the three loading conditions (tension, compression, shear) applied on the samples (*in gray*) as well as their dimensions in the reference undeformed configuration *(bottom)*. *f* represents the average load measured by the load cell. **(B)** Pictures showing a Ge-GA hydrogel stretched during cyclic tension at increasing peak strain levels, and typical stress-strain response, where the mechanical descriptors introduced to quantify the recorded data for each cycle *i* ( $\varepsilon_{i}^{\max}$ , $\varepsilon_{i}^{\mathrm{res}}$ , $P_{i}^{\max}$ , $E_{i}^{t}$ , $W_{i}^{\mathrm{diss}}$ , $W_{i}^{\mathrm{abs}}$ ) have been reported.

**Hygro-mechanical set-up–**Hydrogel samples were tested using an electromechanical tension-compression testing machine (Instron® 5944) equipped with a load cell of ±10 N. All tests were conducted in a thermo-regulated atmosphere (T ≈ 25°C) and at proper hygrometric conditions to prevent samples from air drying: the samples were placed in a chamber (Figure 2A) in which a saturated air flow (≈98–100% RH, quasi-null flow rate Φ_*air*_) was regulated with a heated humidifier (Fisher and Paykel® HC150). The time to reach the prescribed hygrometry was about 30 min, and the capacity of the set-up to maintain it for ≈1 week while preserving the mass and hygro-thermal stability of the samples was also verified (see Supplementary Figure S2A).

During mechanical testing, pictures of the deformed sample were recorded using a high-resolution CCD camera (JAI® BM-500GE, 15 Hz), to quantify its dimensional changes and track cases of sample slippage (Figure 2B). For tensile tests, clamps were coated with sandpaper to facilitate the sample positioning and to minimize its slippage. For compression tests, rectangular compression platens (25 mm length and width) were lubricated by a film of liquid silicone oil, avoiding friction and undesired barrelling effect. For shear tests, plates (16 mm length and 13 mm width) were coated with double-sided adhesive to restrain sample slippage.

**Testing procotol–**Whatever the sample, its mechanical characterization was performed within 24 h–48 h after its manufacturing, following the sequential steps reported below. It should be noted that in this work, the gel samples were not immersed in liquid, neither before nor during the mechanical tests, but rather maintained in a saturated air atmosphere such as that found in the trachea, from their manufacturing until the end of the tests. Each mechanical test was repeated at least 5 times to ensure its reproducibility, using 5 different samples extracted from the same molded plate (Supplementary Figure S3 showing the typical level of scatter in the measurements). For all cases, the loading direction was defined by the vector **e**_*y*_ shown in Figure 2A. In the following, dimensions of undeformed samples along **e**_*y*_ (*resp.* **e**_*x*_, **e**_*z*_) are noted *ℓ*_0_ (*resp.* *w*_0_, *t*_0_).

• Simple tensile tests were first performed on samples cut for an effective length-to-width ratio *ℓ*_0_: *w*_0_ = 5:1, with a gauge length *ℓ*_0_ = 50 mm and a cross-section $S_{0}^{⊥}=$ 20 mm^2^ (see Figure 2A). The cell force *f* signal and the displacement of the machine crosshead *δ* were used to estimate the first Piola-Kirchoff stress $P_{yy}=f/S_{0}^{⊥}$ , as well as the Hencky tensile strain *ɛ*_*yy*_ = ln (1 + *δ*/*ℓ*_0_). The load cell was tared while the sample was subjected to its own weight only. Once mounted between the jaws, the sample was very slightly pre-loaded (*f* ≈ 5.10^–5^ N), and its initial gauge length recalculated accordingly. Then, samples were subjected to N = 4 load-unload cycles with an increasing strain amplitude ( $\varepsilon_{i}^{\max}$ = 0.1, 0.3, 0.5, 0.7, *i* = [1...*N*] – see Figure 2B) and a very low force at each unload phase for the inversion condition (*f* > 5.10^–3^ N). The applied strain rate $\left|{\dot{\varepsilon }}_{yy}|\approx |\dot{\delta }/\ell_{0}\right|$ was parametrically varied from $\approx 10^{-3}\;s^{-1}$ up to $\approx 10^{-1}\;s^{-1}$ . In addition to this first campaign, kinematic conditions were further adjusted to reproduce the tension tests recently conducted on human vocal folds by Cochereau et al. (2020) (*i.e.,* with N = 10, $\varepsilon_{1}^{\max}\;\approx$ 0.1 and $\left|{\dot{\varepsilon }}_{yy}\right|$ = 10^–3^ s^−1^).
   • Simple compression tests were then performed on samples at length-to-width ratio *ℓ*_0_: *w*_0_ = 1:5, with a gauge length *ℓ*_0_ = 2 mm and a cross-section $S_{0}^{⊥}=$ 100 mm^2^ (Figure 2A). Compression stress $P_{yy}=f/S_{0}^{⊥}$ and compression strain *ɛ*_*yy*_ = ln (1 + *δ*/*ℓ*_0_) were recorded during the test. The initial contact between the sample and the top platen was determined once *f* ≈ 5.10^–5^ N (*i.e.,* initial compressive stress ≈O (10^–7^ MPa)). Then, samples were subjected to N = 4 load-unload cycles down to $\varepsilon_{i}^{\min}$ (−0.1, −0.3, −0.5, −0.7) at a strain rate $\left|{\dot{\varepsilon }}_{yy}\right|$ , it being parametrically varied from $\approx 10^{-3}\;s^{-1}$ to 10^–1^ s^−1^. As for tension, the kinematic conditions were also adjusted to reproduce those previously chosen to characterize the compressive response of vocal folds (Cochereau et al., 2020) (*i.e.,* with N = 10, $\varepsilon_{1}^{\min}\;\approx$ -0.2 and $\left|{\dot{\varepsilon }}_{yy}\right|$ = 10^–3^ s^−1^).
• Finally, two samples (*ℓ*_0_ = 10 mm, *w*_0_ = 2 mm, *t*_0_ = 10 mm) were tested together in a symmetrical double-lap configuration ensuring simple shear of the samples along the (**e**_*y*_, **e**_*x*_) plane, as illustrated in Figure 2A (Piollet et al., 2016). Before testing, a slight pre-compression of the samples was imposed (*i.e.,* pre-load of ≈0.05 N in the transverse direction). During the tests, shear stress *P*_*yx*_ = $f/2S_{0}^{//}$ was measured as a function of shear strain *γ*_*yx*_ = *δ*/*w*_0_. Samples were subjected to N = 10 load-unload cycles up to $\gamma_{yx}^{\max}$ = 0.5 at a shear rate $|\dot{\gamma }|=|\dot{\delta }/w_{0}|\approx 10^{-3}s^{-1}$ for comparison with the living tissue database (Cochereau et al., 2020).

Whatever the case, the obtained stress-strain data were quantified by a series of 6 mechanical descriptors displayed in Figure 2B: the peak stress achieved during cycle *i*, noted $P_{i}^{\max}$ (*resp.* $P_{i}^{\min}$ ) if positive (*resp.* negative); the corresponding peak strain, noted $\varepsilon_{i}^{\max}$ (*resp.* $\varepsilon_{i}^{\min}$ ) if positive (*resp.* negative); the tangent modulus assessed at the early stage of the *i* ^th^ unloading phase, $E_{i}^{t}$ , so as to capture instantaneous stiffness of the material; the residual strain occurring at the end of cycle *i*, $\varepsilon_{i}^{\mathrm{res}}$ ; the energy density of the gel, energy stored during the *i* ^th^ load, $W_{i}^{\mathrm{abs}}$ ; the one dissipated after the *i* ^th^ unloading phase, $W_{i}^{\mathrm{diss}}$ and the damping ratio $\eta_{i}=W_{i}^{\mathrm{diss}}/W_{i}^{\mathrm{abs}}$ (Liao et al., 2020).

## Results and discussion

### Effect of cross-linking concentration on the tensile properties of hydrogels

**FIGURE 3:**
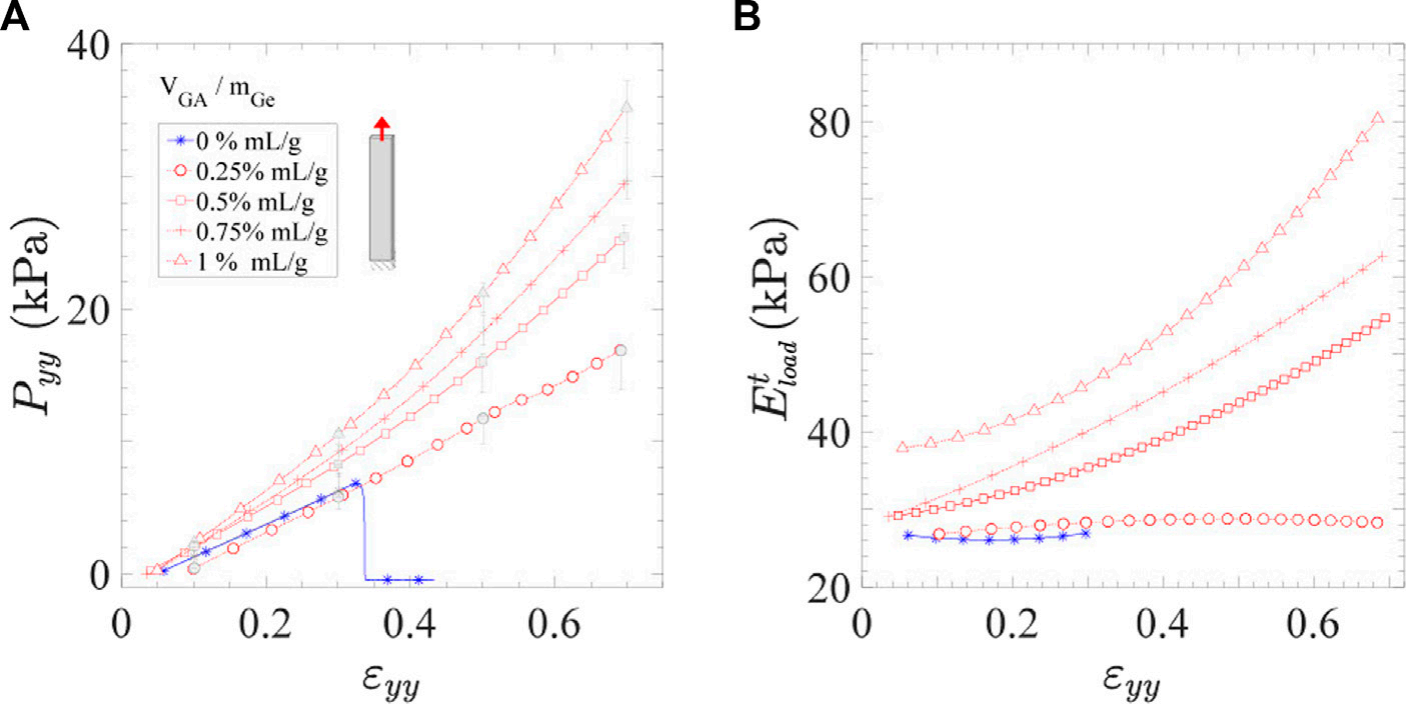
Tensile behavior of Ge (*in blue*) and Ge-GA hydrogels (*in red*) with various concentrations of cross-linker: **(A)** Stress-strain curves (during the only loading part of last cycle) and **(B)** corresponding tangent modulus $E_{\mathrm{load}}^{t}$ as a function of *ɛ*_*yy*_. $|{\dot{\varepsilon }}_{yy}|\approx$ 10^–2^ *s* ^−1^.

The tensile responses of Ge and Ge-GA hydrogels recorded during the last loading at $|{\dot{\varepsilon }}_{yy}|\approx 10^{-2}\;s^{-1}$ up to $\varepsilon_{i}^{\max}$ = 0.7 are displayed in Figure 3, together with the evolution of the tangent moduli $E_{\mathrm{load}}^{t}=dP_{yy}/d\varepsilon_{yy}$ with *ɛ*_*yy*_. Ge samples demonstrate a quasi-linear tensile response with a nearly constant tangent modulus $E_{\mathrm{load}}^{t}$ ≈ 27 kPa, up to failure which occurs when *ɛ*_*yy*_ ≈ 0.32 (see Figure 3A). By contrast, the addition of chemical cross-linking during the manufacture of the gels results in: (i) improved ductility, in that Ge-GA samples do not break at *ɛ*_*yy*_ = 0.7, even at the lowest GA concentrations; (ii) improved tensile strength, with higher stress levels registered from moderate GA concentrations (*i.e.,* *V*_*GA*_/*m*_*Ge*_ ≥ 0.5% mL/g). By comparing such Ge-GA mixtures to neat gelatin at *ɛ*_*yy*_ ≈ 0.3 for instance, the ratio of peak stresses $P_{i}^{\max}$ ranges from 1.1 to 1.6. By further comparing the most and the least concentrated GA hydrogels at higher strains (*ɛ*_*yy*_ ≈ 0.7), this ratio rises up to about 2.1. (iii) induced non-linearity of the stress-strain response and strain hardening of tangent moduli once *V*_*GA*_/*m*_*Ge*_ ≥ 0.5% mL/g, as clearly evidenced in Figure 3B. This critical threshold of GA concentration needed to enhance the mechanical properties of Ge hydrogels is also highlighted in Supplementary Figure S4 for all cycles applied from $\varepsilon_{i}^{\max}=$ 0.1 to 0.7. In the following, due to casting difficulties and undesired cytotoxic effects likely to occur at higher GA concentrations (Bigi et al., 2001), focus is made on the cross-linked hydrogels with this critical degree of cross-linking (*V*_*GA*_/*m*_*Ge*_ = 0.5% mL/g). Finally, although contrary qualitative trends were observed by Bigi et al. (2001) with, in particular, the extensibility of Ge films not favored by increasing GA concentration, our results are consistent with those obtained by Farris et al. (2011) in gelatin-pectin-glycerol films cross-linked with GA.

### Mechanics of hydrogels in tension and compression

**FIGURE 4:**
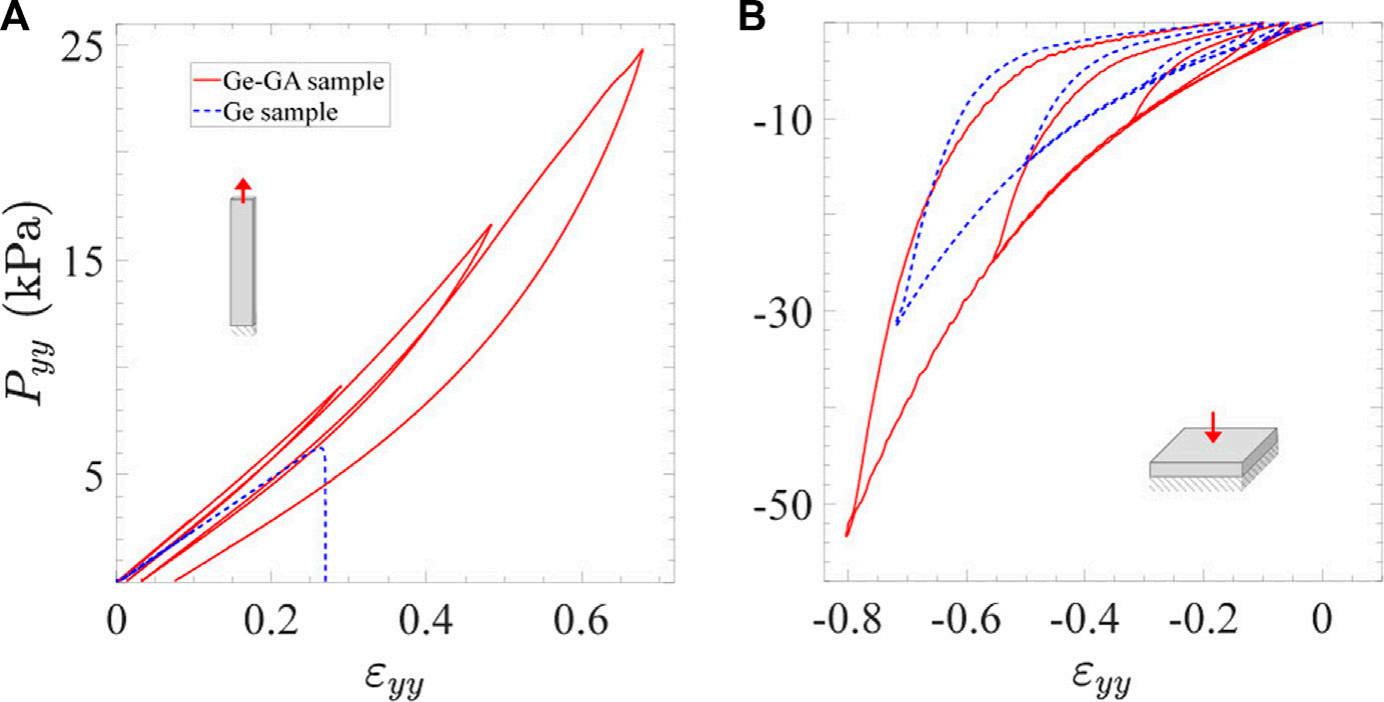
Stress-strain response of Ge samples and Ge-GA samples (*V*_*GA*_/*m*_*Ge*_ = 0.5% mL/g) measured in cyclic tension **(A)** and compression **(B)** at $|{\dot{\varepsilon }}_{yy}|\approx$ 10^–2^ *s* ^−1^.

**FIGURE 5:**
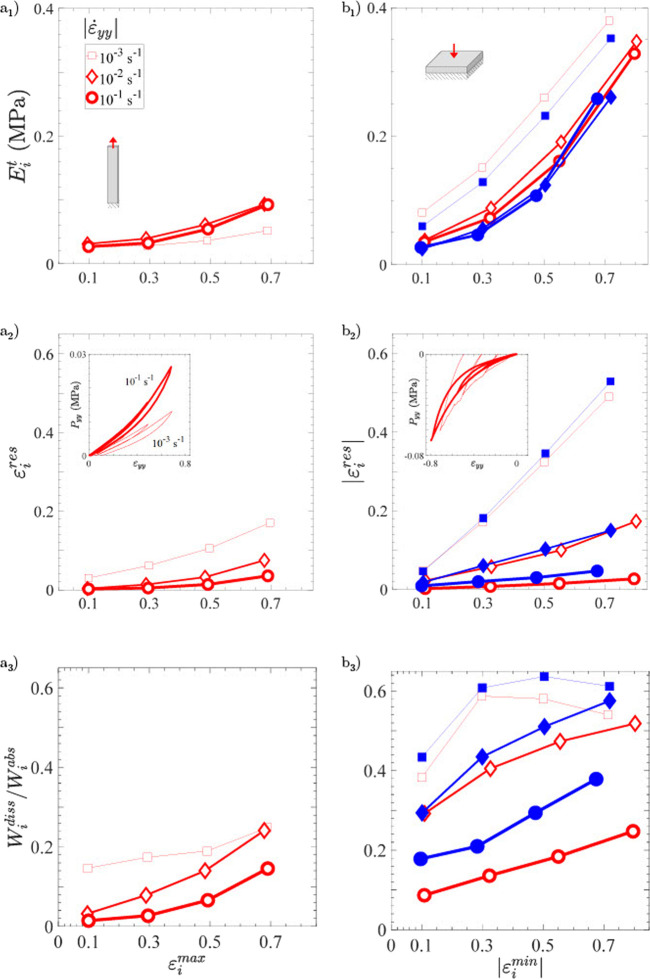
Mechanical descriptors of Ge (*in blue*, *filled symbols*) and Ge-GA hydrogels (*in red*, *blank symbols*) according to the loading type and the applied strain rate: **(a1)** tangent modulus, **(a2)** residual strain per cycle, and **(a3)** damping ratio per cycle in function of the cycle strain amplitude in tension. **(b1, b2, b3)** Same as **(a1, a2, a3)** but in compression.

In this section, the mechanics of the previously selected cross-linked hydrogel (*V*_*GA*_/*m*_*Ge*_ = 0.5% mL/g) is compared to that of neat gelatin in tension and compression at various strain rates $\left|{\dot{\varepsilon }}_{yy}\right|$ (from 10^–3^ s^−1^ to 10^–1^ s^−1^). Figure 4 shows typical stress-strain curves obtained with Ge and Ge-GA samples subjected to progressive cycles from $\varepsilon_{i}^{\max}$ = 0.1 to 0.7 in tension and compression at $|{\dot{\varepsilon }}_{yy}|\;\approx$ 10^–2^ s^−1^. The strain evolution of the mechanical descriptors for each cycle is illustrated in Figure 5 (*diamond symbols*), and further detailed in Table 1. Note that the descriptors of neat Ge (*in blue*) at largest strains were only calculated in compression mode, as it was not able to sustain tensile strains beyond 0.35 over the whole database.

**TABLE 1:** Average mechanical descriptors recorded for Ge and Ge-GA hydrogels at various strain rates $\left|{\dot{\varepsilon }}_{yy}\right|$ , applied absolute strain |*ɛ*_*yy*_|, and GA concentrations.^†^|*ɛ*_*yy*_| is equivalent to $\varepsilon_{i}^{\max}$ in tension and - $\varepsilon_{i}^{\min}$ in compression.^††^× corresponds to cases of failure.

			Tension				Compression			
$\left|{\dot{\varepsilon }}_{yy}\right|$ (s^−1^)	*V_*GA*_/m_*Ge*_* (% mL/g)	|*ɛ*_*yy*_|^†^	$E_{i}^{t}$ (kPa)	$\varepsilon_{i}^{\mathrm{res}}$	*η*_*i*_	$P_{i}^{\max}$ (kPa)	$E_{i}^{t}$ (kPa)	$\varepsilon_{i}^{\mathrm{res}}$	*η*_*i*_	$P_{i}^{\min}$ (kPa)
10^–1^	0	0.1	25 ^×^	0.01	0.07	2	27	−0.01	0.18	−2
		0.3	25	0.01	0.09	7	46	−0.02	0.21	−8
		0.5	×	×	×	×	107	−0.03	0.29	−18
		0.7	×	×	×	×	258	−0.05	0.38	−39
	0.5	0.1	27	0.00	0.01	1	35	0.00	0.14	−3
		0.3	33	0.00	0.03	7	72	−0.01	0.14	−12
		0.5	54	0.01	0.07	15	161	−0.01	0.18	−30
		0.7	93	0.03	0.14	25	328	−0.03	0.25	−68
10^–2^	0	0.1	27	0.00	0.04	2	27	−0.02	0.30	−2
		0.3	×	×	×	×	56	−0.06	0.43	−6
		0.5	×	×	×	×	124	−0.10	0.51	−15
		0.7	×	×	×	×	260	−0.15	0.58	−31
	0.5	0.1	31	0.00	0.03	3	38	−0.02	0.29	−3
		0.3	39	0.01	0.08	9	88	−0.06	0.40	−10
		0.5	61	0.03	0.14	16	191	−0.10	0.47	−25
		0.7	93	0.07	0.24	25	347	−0.17	0.52	−53
10^–3^	0	0.1	21	0.02	0.30	1	59	−0.04	0.43	−3
		0.3	×	×	×	×	128	−0.18	0.61	−12
		0.5	×	×	×	×	231	−0.34	0.64	−25
		0.7	×	×	×	×	352	−0.53	0.61	−47
	0.5	0.1	27	0.00	0.15	2	81	−0.05	0.38	−4
		0.3	28	0.00	0.17	6	151	−0.17	0.59	−15
		0.5	36	0.01	0.19	9	259	−0.32	0.58	−32
		0.7	52	0.03	0.25	13	379	−0.49	0.54	−60

*General trends–*Compared to tension, neat gelatin is able to withstand much larger strains and stress levels in compression without breaking (Figure 4). Typically, the ultimate tensile strength of Ge samples was measured around 6 kPa, whereas the compressed samples were able to endure ≈5 times higher stress levels at $\varepsilon_{i}^{\min}=-0.7$ . Looking in more detail at the compressive response of gelatin while cycling, the progressive load-unload sequences yield here to a non-linear mechanical response with a strain hardening, and a stress hysteresis with non-negligible residual strain $\varepsilon_{i}^{\mathrm{res}}$ after unloading (up to 0.15 at the last cycle). Figures 5(b1–b3) clearly highlights the non-linear increase of $E_{i}^{t}$ , $\varepsilon_{i}^{\mathrm{res}}$ , $W_{i}^{\mathrm{diss}}$ with *ɛ*_*yy*_.

Regarding the Ge-GA samples, let us first note that the orders of magnitude of the nominal stresses we obtained in compression are in line with measurements recently performed by Michelini et al. (2020) on a similar gelatin (from porcine skin, 300 g Bloom, Type A), cross-linked by GA and tested at a comparable strain rate down to $\varepsilon_{i}^{\min}=-0.3$ , although at 37°C under hydrated conditions. Then, while the addition of GA in gelatin has a strong impact in tension in terms of ductility (see previous section and Figure 4A), the comparison between the properties of Ge and Ge-GA hydrogels in compression gives much closer qualitative and quantitative trends (see Figure 4B). This is particularly demonstrated in Figures 5b1–b3, where rather similar (*blue* vs. *red*) values are obtained for all (un)cross-linked samples, in terms of tangent moduli $E_{i}^{t}$ , residual strains $\varepsilon_{i}^{\mathrm{res}}$ or dissipated energy per cycle $W_{i}^{\mathrm{diss}}$ . Finally, note that the non-linear increase of $E_{i}^{t}$ , $\varepsilon_{i}^{\mathrm{res}}$ and $W_{i}^{\mathrm{diss}}$ with the strain is also observed for Ge-GA samples stretched in tension (Figures 5a1–a3). However, it remains much less pronounced than in compression, thereby implying a lower degree of non-linearity, weaker hysteretic cyclic response and more reversible deformations in tensile mode.

*Effect of strain rate–* Figure 5 and Table 1 show the evolution of $E_{i}^{t}$ , $\varepsilon_{i}^{\mathrm{res}}$ , *η*_*i*_ $=W_{i}^{\mathrm{diss}}/W_{i}^{\mathrm{abs}}$ obtained for Ge(-GA) hydrogels with $\left|{\dot{\varepsilon }}_{yy}\right|$ in both tension and compression. The corresponding stress-strain curves measured for all cases are reported in Supplementary Figure S5. First of all, the overall stress-strain response of the hydrogels, and thus its mechanical descriptors ( $E_{i}^{t}$ , $\varepsilon_{i}^{\mathrm{res}}$ , *η*_*i*_) remain rather close when deformed at $|{\dot{\varepsilon }}_{yy}|\approx \;10^{-2}s^{-1}$ or 10^−1^s^−1^ (see Figure 5; Supplementary Figure S5). In any case, for both loading modes, the measured changes are consistent with the expected responses of standard viscoelastic materials: the higher the loading rate, the higher the stress level and stiffness, and the lower the residual strain and dissipated energy (*e.g.,* see quasi-null $\varepsilon_{i}^{\mathrm{res}}$ in Figure 5b_2_ and lowest ratio *η*_*i*_ in Figure 5b_3_). These standard trends have been previously observed in many living soft tissues (Cochereau et al., 2020), elastomers, gellan gum gels (Nakamura et al., 2001; Teratsubo et al., 2002; Sharma and Bhattacharya, 2014), or hydrogels with reversible hydrophobic associations during uniaxial extension (Wang et al., 2019). Such macroscale properties of polymers are often ascribed to time-dependent nanostructural rearrangements (*e.g.,* unfolding of entangled molecular chains overcoming friction from other chains, (un)binding, deformation or rupture of cross-links) and/or fluid motion (Bot et al., 1996; Sharma and Bhattacharya, 2014; Cacopardo et al., 2019; Huang et al., 2019; Wang et al., 2019), which are not instantaneous processes, but instead require some time to occur.

Astonishingly, these common trends are not all fulfilled for the lowest strain rate $|{\dot{\varepsilon }}_{yy}|\approx \;10^{-3}s^{-1}$ , regardless of the type of loading and material.

• Among the expected trends, residual strains are strongly increased, shifting from 0.15 (for $|{\dot{\varepsilon }}_{yy}|\approx$ 10^−2^s^−1^) to 0.53 for example for compressed gelatin after unloading at $\varepsilon_{i}^{\min}=-0.7$ . This inelastic effect is accentuated cycle by cycle with the amplitude of the applied strain whatever the loading mode, and all the more marked in compression (see Figure 5a2 vs. Figure 5b2). Likewise, the damping properties of the gels are particularly enhanced at this slowest speed, for both tensile and compressive modes: whatever the case, the ratio of dissipated to absorbed elastic energy after deformation of hydrogels, $\eta_{i}=W_{i}^{\mathrm{diss}}/W_{i}^{\mathrm{abs}}$ , remains higher than that obtained at higher strain rates (see Figures 5a3, b3. Typically, with respect to the highest (*resp.* intermediate) strain rate $\left|{\dot{\varepsilon }}_{yy}\right|$ , the relative increase of $W_{i}^{\mathrm{diss}}/W_{i}^{\mathrm{abs}}$ measured during compression ranges from 118% up to 341% (resp. 4% up to 45%) depending on the applied strain.
• However, in a strange and still unexplained way, the instantaneous stiffness of the hydrogels after unloading is altered in reverse trends in tension and compression: in tension, the gel viscoelasticity yields well to a moderate decrease of stress levels (Supplementary Figure S5 (a_1,2_)) and tangent moduli $E_{i}^{t}$ (Figure 5a_1_) compared to the highest rates, especially for $\varepsilon_{i}^{\max}>0.3$ . However, a very singular behavior is evidenced in compression, showing both higher stress levels (Supplementary Figure S5 (b_1,2_)) and tangent moduli $E_{i}^{t}$ (Figure 5b_1_) from the early deformation stages $\left(\varepsilon_{i}^{\min}\leq -0.1\right)$ .

To our knowledge, we have not seen this type of behavior before in the literature. Focusing more specifically on gelatin gels, a few studies have already reported the strain rate sensitivity of gels in compression (Kwon and Subhash, 2010; Forte et al., 2015). In particular, for the three strain rates studied in our database, Forte et al. (2015) observed that gelatin gels (beef origin) exhibit a strong rate dependent failure response (with both ultimate stress and strain rising with the applied rate). This rate effect was reproduced by modelling the gel as a poroelastic material with water flow through the porous solid polymer network. By simulating monotonic compression tests on a neat gelatin sample assumed to be fully saturated, their predictions show that solid matrix stresses increase while pore pressure decreases as the strain rate decreases. At low strain rates (typically 10^–3^ s^−1^), the liquid is expected to easily flow into the solid matrix network (or even leave the sample), thus contributing very little to the gel’s load resistance and failure. Thus, in the critical case of gel cracking and liquid migration, we would expect an increase in stress levels and gel stiffness at low strain rates. However, this consolidation scenario is not viable in our case for no cracks or water flow out of the sample were observed during the experiments. The origin of the singular behavior evidenced in our results remains an open question.

### Comparison with human vocal folds

To finally quantify the relevance of gelatin-based hydrogels as biomimetic candidates, the cyclic and finite strains mechanics of the Ge-GA hydrogels processed above is compared with that of human vocal folds. The target mechanical behavior of native tissues was chosen as characterized *ex vivo* by Cochereau et al. (2020) under multiple loadings relevant in phonation, *i.e.,* longitudinal tension, transverse compression and longitudinal shear. Over the whole database, we determined the Ge-GA candidate whose mechanical properties best reproduced the reference data on average for these three loading modes. The best candidate was obtained for the concentration of cross-linker *V*_*GA*_/*m*_*Ge*_ = 0.5% mL/g.

**FIGURE 6:**
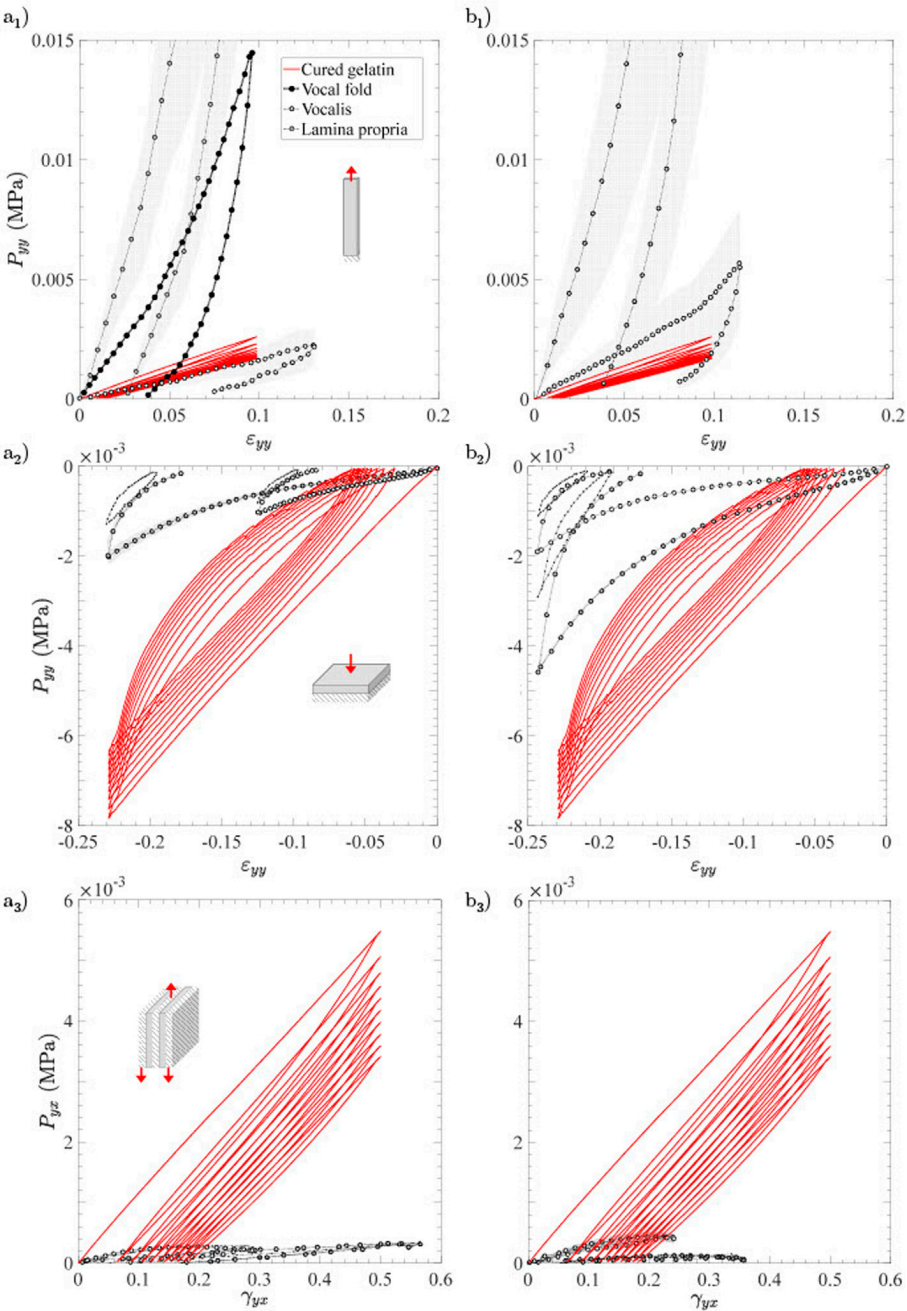
Typical stress-strain curves of the Ge-GA hydrogel with *V*_*GA*_/*m*_*Ge*_ = 0.5% mL/g (*in red*), and those measured on one human vocal fold and its main sublayers (*lamina propria*, *vocalis*) when subjected successively to 10 load-unload cycles in **(a1)** longitudinal tension, **(a2)** transverse compression, **(a3)** longitudinal shear. **(b1, b2, b3)** Same as **(a1, a2, a3)** but for another donor. *Source:* Adapted from Cochereau et al. (2020).

Figure 6 shows typical stress-strain curves obtained after subjecting the selected hydrogel to 10 load-unload cycles in tension, compression and shear (*in red*), using the same geometrical and kinematical conditions chosen for the native tissue (Cochereau et al., 2020). Reference data obtained on vocal folds and their major layers (the *lamina propria*, *i.e.,* the upper loose connective tissue, and the *vocalis* muscle below) dissected from two healthy human larynges are reported in Figure 6: graphs (a_1_, a_2_, a_3_) give data from a 79-year-old male donor (height 1.70 m, weight 65 kg), whereas graphs (b_1_, b_2_, b_3_) refer to a 79-year-old female donor (height 1.60 m, weight 45 kg). Regarding these biological targets, note that only the 1^*st*^ and 10^th^ cycle are displayed for the sake of clarity. In addition, gray corridors represent stress-data uncertainty (1^*st*^ cycle only) induced by the estimation of the sample cross section.

Firstly, it is important to remind that both *lamina propria* and *vocalis* can be seen as 3D incompressible composite structures made of a gel-like matrix reinforced by a network of collagen fibers, with wavy shapes and preferred orientations at rest (Kelleher et al., 2013; Miri et al., 2013; Bailly et al., 2018; Terzolo et al., 2022). Knowing that, our results show that the average properties of the Ge-GA hydrogel (stiffness and strength) are quite comparable (albeit higher) to those of the two vocal-fold layers in transverse compression and longitudinal shear, *i.e.,* under loading conditions where fibers unfolding, tension and rotation are limited, while the mechanical contribution of the isotropic matrix is much more critical (Terzolo et al., 2022). Higher quantitative discrepancies are also found with the tensile response of the entire fold and its upper layer, due to the progressive recruitement and reorientation of the collagen fibers towards the load direction in this case (Min et al., 1995; Gasser et al., 2006; Terzolo et al., 2022). For instance, at a strain of 0.1 (absolute value), the stress level in the Ge-GA sample is about 17 times lower than that achieved in the *lamina propria* in tension (mean value on both donors), while it is about 5 times higher in compression and 4 times higher in shear. Note that in tension, deviations from the *vocalis* muscle become far less pronounced, because the muscle fibers are straighter and softer than the collagen fibers of the *lamina propria* at rest (Bailly et al., 2018; Terzolo et al., 2022).

In the end, despite its isotropy, the chosen Ge-GA hydrogel proves to be a first and rather basic solution to approximate the average behavior of the *vocalis* and the *lamina propria* for the three loading modes. While it is thus able to mimic the tensile behavior of the *vocalis* fairly well, it fails to mimic quantitatively that of the *lamina propria* due to the strong tissue anisotropy. Embedding a fibrous reinforcement in the hydrogel or inducing a suitable nanostructuration using freeze-drying techniques (Martoïa et al., 2016; Gupta et al., 2018; Grenier et al., 2019) should allow to approach the J-shaped anisotropic target response in tension, without further stiffening the current properties in compression and shear.

## Conclusion

The mechanics of hydrogels made of neat or cross-linked gelatin with parametric concentrations of glutaraldehyde were characterized under tension, compression and shear, upon finite strains and over 3 decades of strain rates. In summary, the collected database has highlighted several original outcomes:

• a critical concentration of cross-linker is needed to enhance the mechanical strength, stiffness and ductility of neat gelatin in tension (*V*_*GA*_/*m*_*Ge*_ ≥ 0.5% mL/g);
• compared to tension, neat gelatin is able to withstand much larger strains and stress levels in compression without breaking, and the mechanics of neat and cross-linked hydrogels are rather close in that mode;
• whatever the type of loading and material, a very specific strain-rate sensitivity of the gels is evidenced. In particular, a drastic change in mechanical behavior is observed for the lowest strain rate at 10^–3^ s^−1^ compared to the upper 2 decades, showing both higher stress levels and tangent moduli in that case;
• finally, to mimic the tension, compression and shear responses of the vocal-fold fibrous tissues, the cross-linked hydrogels developed in this work prove to be rather relevant candidates despite their isotropy.

Developments are still needed to better understand these multiaxial mechanical properties evidenced at the macroscale. In particular, information about the internal network structure of the various gels such as their pore topology should be explored, using *ante-*/*post-mortem* micro-imaging techniques (Marmorat, 2016). Regarding the target application, the introduction of a suitable structuration in the proposed hydrogels should now be conducted to mimic the J-shaped anisotropic tensile response of the vocal folds.

## Data Availability

The raw data supporting the conclusion of this article will be made available by the authors, without undue reservation.
