# Supplementary material for: Mechanics of gelatin-based hydrogels during finite strain tension, compression and shear
Source: Front Bioeng Biotechnol. 2023 Jan 12;10:1094197. doi: 10.3389/fbioe.2022.1094197 (PMC9877534; doi:10.3389/fbioe.2022.1094197)
Supplement: Supplementary file 1 [file DataSheet1.pdf]

Supplementary Figures

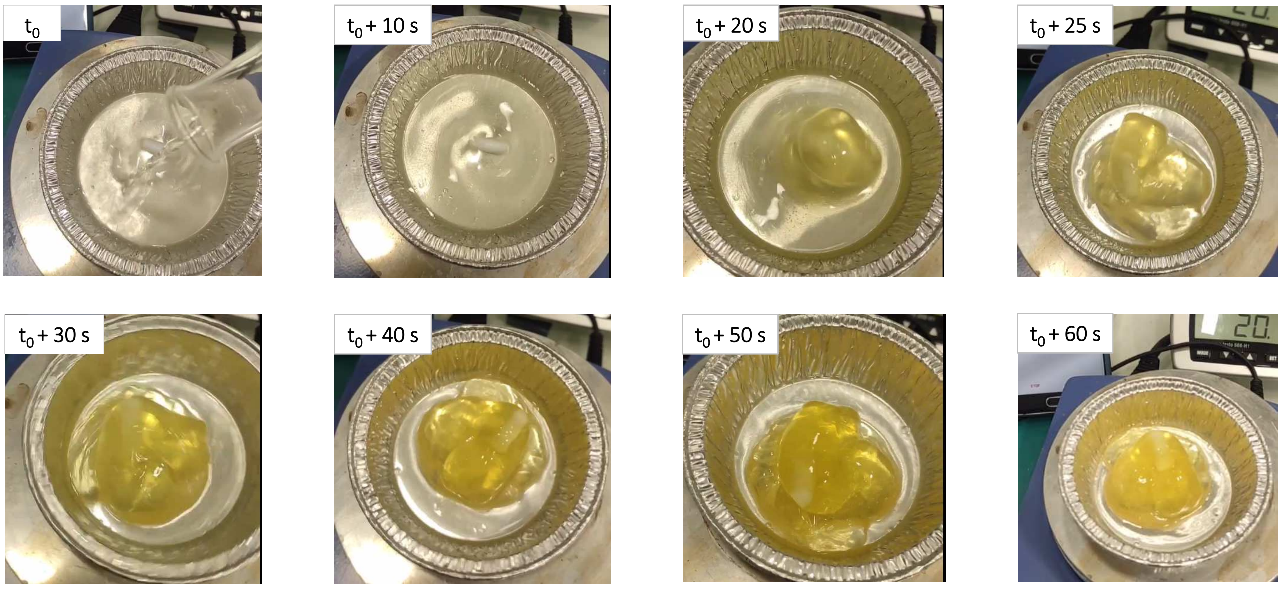

Figure S1. Kinematics of the cross-linking reaction during the elaboration of Ge-GA hydrogels in case $V_{GA}/m_{Ge} > 1$ % mL/g.

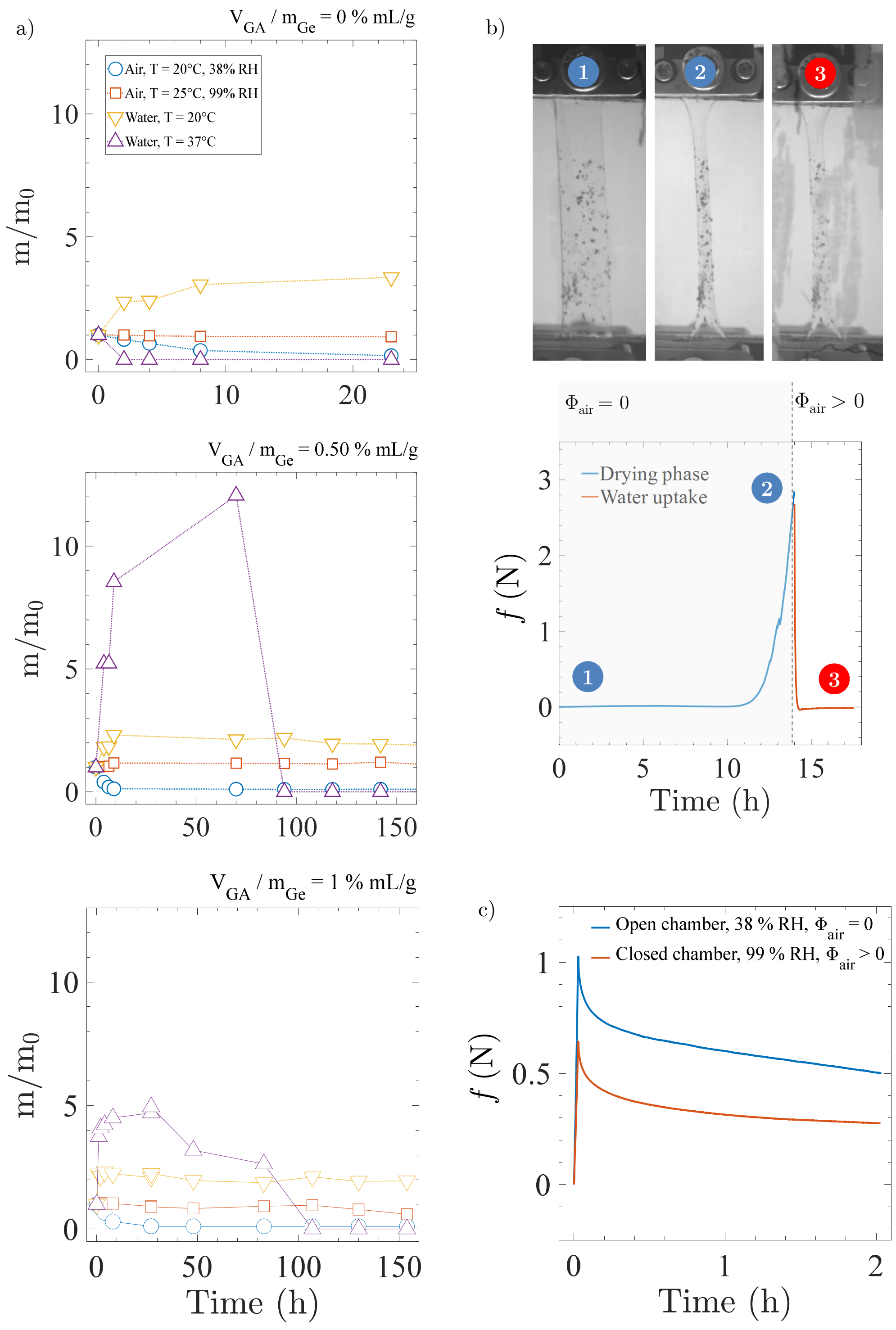

Figure S2. Stability of Ge-GA hydrogels assessed in various hygro-thermal environments. a) Mass evolution of a typical Ge-GA sample in function of time for three different V_{GA}/m_{Ge} ratios (*from top to bottom*) : 0.00%, 0.50% and 1.00% mL/g. b) Typical time-evolution of the axial load measured while maintaining a humidified Ge-GA sample (99 % RH) between the tensile jaws, once $\Phi_{air} = 0$ (*drying phase in gray zone from time ① to time ②*) and $\Phi_{air} > 0$ (*water uptake from time ② to time ③*). c) Typical stress relaxation behaviour of Ge-GA hydrogels after a pre-strain of 70% with $|\dot{\varepsilon}_{yy}| \approx 10^{-2}\ s^{-1}$, placed in two distinct hygro-thermal conditions : (*in blue*) T = 20 °C, 38 % RH, $\Phi_{air} = 0$; (*in red*) T = 25 °C, 99 % RH, $\Phi_{air} > 0$.

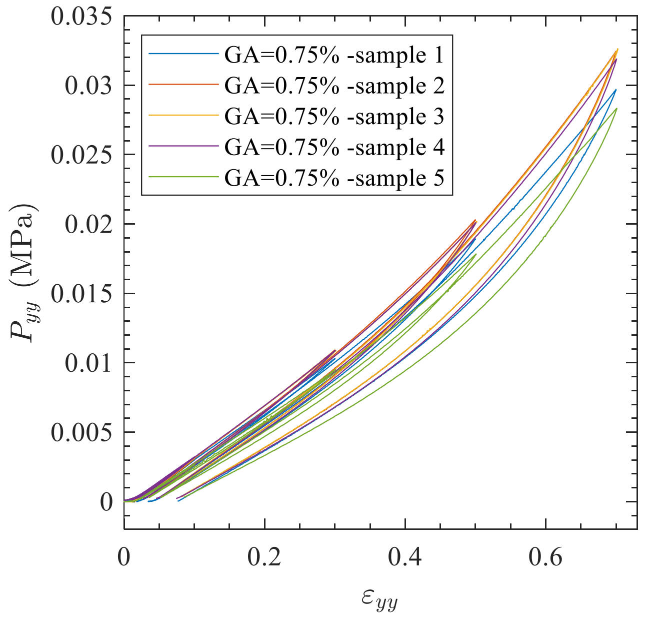

Figure S3. Typical stress-strain repeatability obtained for the cyclic tensile test performed on Ge-GA hydrogels over 5 measurements. Illustrative case of the Ge-GA hydrogel with $V_{GA}/m_{Ge} = 0.75$ % mL/g.

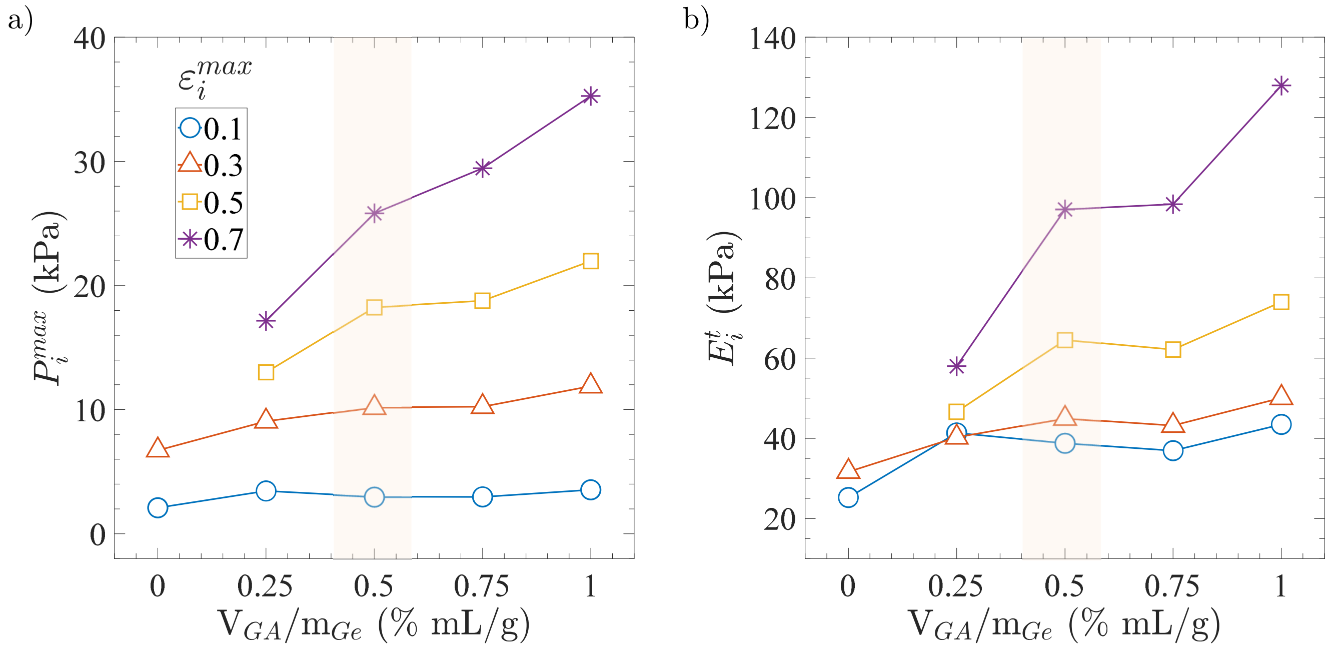

Figure S4. Influence of the cross-linker concentration (V_{GA}/m_{Ge}) on two mechanical descriptors of Ge-GA samples during tensile cyclic loading, as a function of maximum strain amplitude ε_i^{max} : a) peak stress achieved during cycle i, P_i^{max}; b) tangent modulus at the early unloading phase of each cycle, E_i^t. $|\dot{\varepsilon}_{yy}| \approx 10^{-2}\ s^{-1}$. Red-colored area corresponds to the critical degree of cross-linking.

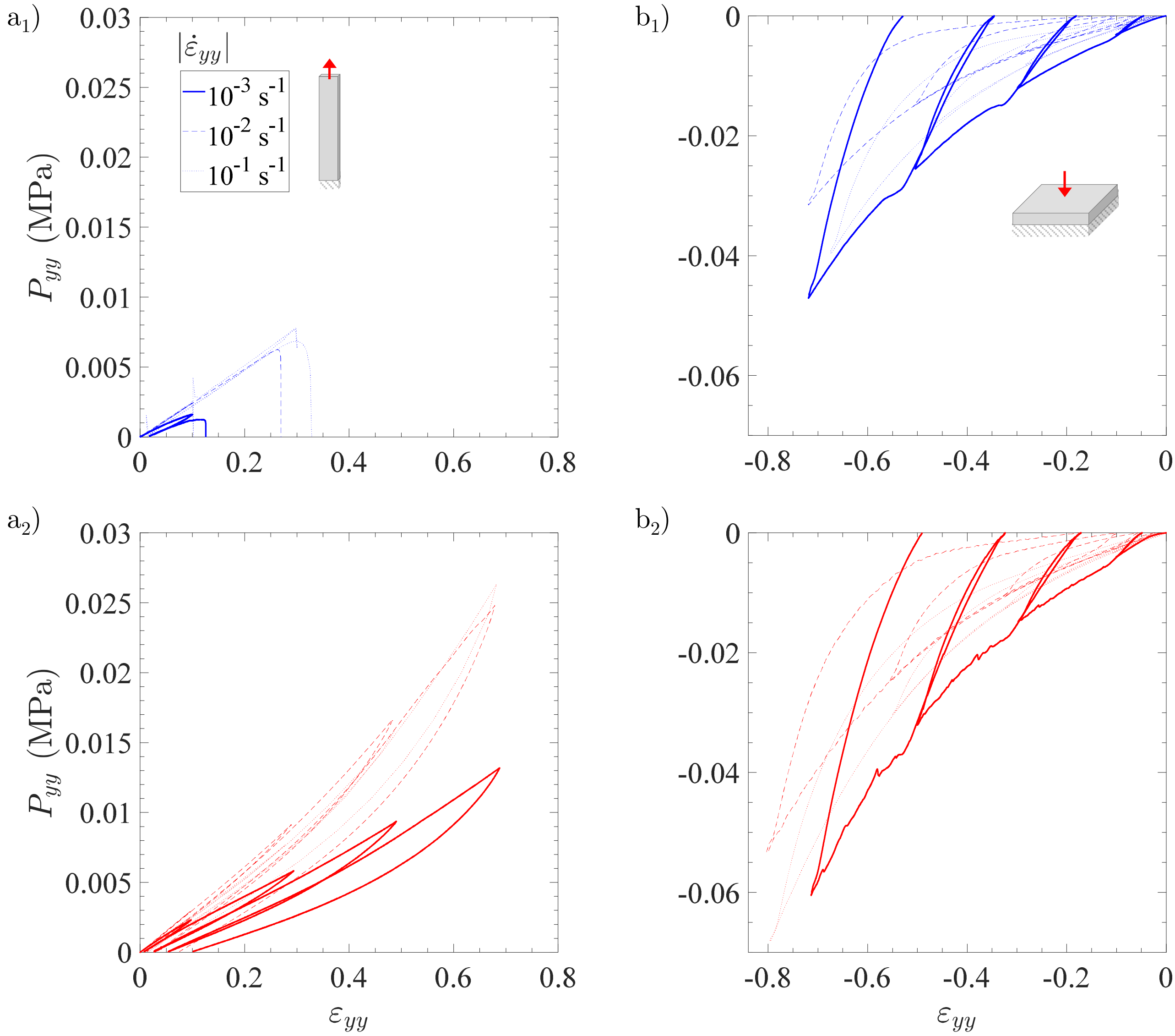

Figure S5. (a_1, a_2) Tensile stress-strain response of Ge samples (*in blue*; V_{GA} = 0 mL) and Ge-GA samples (*in red*; $V_{GA}/m_{Ge} = 0.5$ % mL/g) for three orders of magnitude of strain rate $|\dot{\varepsilon}_{yy}|$. (b_1, b_2) same as (a_1, a_2) but in compression.
